# Hematological Composite Scores in Patients with Inflammatory Bowel Disease

**DOI:** 10.3390/jcm12237248

**Published:** 2023-11-23

**Authors:** Marta Carrillo-Palau, Belén Vera-Santana, Andrea Morant-Domínguez, Alejandro Hernández-Camba, Laura Ramos, Inmaculada Alonso-Abreu, Noemi Hernández Álvarez-Buylla, Laura Arranz, Milagros Vela, Manuel Hernández-Guerra, Cristina Gómez-Moreno, Miguel Á. González-Gay, Iván Ferraz-Amaro

**Affiliations:** 1 Division of Gastroenterology, Hospital Universitario de Canarias, 38320 Tenerife, Spain; mcarpal@gobiernodecanarias.org (M.C.-P.); bversanq@gobiernodecanarias.org (B.V.-S.); amordom@gobiernodecanarias.org (A.M.-D.); lrlopez@gobiernodecanarias.org (L.R.); ialoabr@gobiernodecanarias.org (I.A.-A.); nheralv@gobiernodecanarias.org (N.H.Á.-B.); mhernand@ull.edu.es (M.H.-G.); 2 Division of Gastroenterology, Hospital Universitario de Nuestra Señora de la Candelaria, 38010 Tenerife, Spain; ahercamxl@gobiernodecanarias.org (A.H.-C.); larrher@gobiernodecanarias.org (L.A.); mvelgon@gobiernodecanarias.org (M.V.); 3 Fundación Jiménez Díaz School of Nursing of Madrid, Autonomous University of Madrid, 28040 Madrid, Spain; cgomezm@fdj.es; 4 Division of Rheumatology, Instituto de Investigación Sanitaria Fundación Jiménez Díaz, 28040 Madrid, Spain; 5 Department of Medicine, University of Cantabria, 39005 Santander, Spain; 6 Division of Rheumatology, Hospital Universitario de Canarias, 38320 Tenerife, Spain; 7 Department of Internal Medicine, Universidad de La Laguna (ULL), 38200 Tenerife, Spain

**Keywords:** inflammatory bowel disease, systemic immune-inflammatory index, hematological inflammatory scores

## Abstract

The neutrophil-to-lymphocyte ratio (NLR), platelet-to-lymphocyte ratio (PLR), monocyte-to-lymphocyte ratio (MLR), and the systemic immune-inflammatory index (SIRI, neutrophils × monocytes/lymphocytes) have been identified as potential inflammatory biomarkers. In this work we aimed to analyze whether the hematological composite scores differ between inflammatory bowel disease (IBD) patients and healthy controls, and if they are related to disease activity. A total of 197 IBD patients—130 Crohn’s (CD) disease and 67 ulcerative colitis (UC)—and 208 age- and sex-matched healthy controls were enrolled. C-reactive protein and fecal calprotectin were assessed. Multivariable linear regression analysis was executed. After adjustment, NLR and PLR, but not SIRI and MLR, were significantly higher in IBD patients compared to controls. C-reactive protein and SIRI and NLR were correlated in IBD patients. However, fecal calprotectin was not related to any of these blood scores. Furthermore, disease activity parameters were not associated with any of the blood composite scores in both CD and UC patients. In conclusion, NLR and PLR, but not SIRI and MLR, are independently higher in IBD patients compared to controls. However, the four hematological scores are not related to disease activity in either CD or UC patients. Based on these results, blood-based inflammatory scores may not serve as subrogated biomarkers of disease activity in IBD.

## 1. Introduction

The Systemic Inflammation Response Index (SIRI) is a novel prognostic marker that relies on the relative proportions of peripheral neutrophils, monocytes, and lymphocytes, calculated by multiplying the neutrophil count by the monocyte count and then dividing by the lymphocyte count [1]. This score is part of the group of other hematological scores previously described, such as the neutrophil-lymphocyte ratio (NLR) [2], the platelet-lymphocyte ratio (PLR) [3], and the lymphocyte-monocyte ratio (LMR) [4]. These are regarded as markers of inflammation because white blood cells and platelets are typically present in acute and chronic inflammatory settings, where they release cytokines, proteases, angiogenic factors, and chemokines [5]. In recent years, these scores have generated interest and have gained relevance because they have been described as relating to or predicting certain outcomes in chronic inflammatory, autoimmune [6,7,8], and cardiovascular diseases [9], as well as in cancer [10] or infections [11].

Inflammatory bowel disease (IBD) comprises two main disorders: ulcerative colitis (UC) and Crohn’s disease (CD). While UC affects the colon, CD can affect any component of the gastrointestinal tract from the mouth to the perianal area. Both are considered inflammatory conditions characterized by relapsing and remitting episodes. Inflammation in UC is limited to the mucosal layer of the colon whereas CD is characterized by transmural inflammation and by skip areas of involvement. Recent findings indicate that subjective assessments of disease activity in IBD may be misleading [12]. Moreover, while objective inflammation markers are closely linked to significant long-term results, they frequently necessitate invasive and costly procedures like ileocolonoscopy and cross-sectional imaging techniques involving computed tomography or magnetic. For this reason, in practice, serum or fecal markers such as C-reactive protein (CRP) and fecal calprotectin are used as measures of intestinal inflammation. However, they are not specific for IBD [13,14], cut-off points have not been defined for both that determine activity [15,16], and their diagnostic or predictive capacity for relapse is doubtful [17,18,19]. For this reason, the challenge persists for locating inflammation markers that are more precise, sensitive, and adaptive, with the aim of enhancing IBD management.

In the present work we sought to determine if blood composite scores differ between IBD patients and controls. In a second step, we aimed to analyze whether these scores are related to acute phase reactants, fecal calprotectin or specific disease activity scores in patients with IBD, including UC and CD. If these scores were related to the activity of the disease, they could be proposed as surrogate biomarkers of the disease and, perhaps, be used as such in clinical practice.

## 2. Materials and Methods

### 2.1. Study Participants

A cross-sectional study was conducted, involving 197 consecutive patients diagnosed with IBD and 208 age-matched controls, all of whom were 18 years of age or older. IBD individuals were under the care of gastroenterologists and received periodic follow-ups at gastroenterology outpatient clinics. Inclusion criteria were a diagnosis of IBD based on clinical, endoscopic, and histological criteria with a disease duration of ≥1 year. Exclusion criteria for both groups included a history of cancer, any other inflammatory or autoimmune chronic disease, or evidence of active infection (because this could lead to upregulation of different blood cell types). The control group consisted of individuals from the community recruited by general practitioners in primary health centers. The study protocol was approved by the Institutional Review Committees at Hospital Universitario de Canarias and Hospital Universitario Nuestra Señora de La Candelaria, both located in Spain, and all participants provided written informed consent (approval no. CHUC_2019_103). Research involving human subjects adhered to the principles of the Helsinki Declaration.

### 2.2. Data Collection

Questionnaires regarding clinical history were conducted in both IBD patients and control groups to evaluate cardiovascular risk factors and medication usage. Hypertension was characterized as having a systolic blood pressure exceeding 140 mmHg or a diastolic blood pressure exceeding 90 mmHg. Disease activity in patients with CD was determined using two measures: the Crohn’s Disease Activity Index (CDAI) and the Harvey-Bradshaw Index (HBI) [20]. Disease activity in UC was calculated through the partial Mayo Clinic score [21]. Dyslipidemia was determined based on meeting one or more of the following criteria: total cholesterol exceeding 200 mg/dL, triglyceride levels exceeding 150 mg/dL, HDL-cholesterol lower than 40 mg/dL in men or less than 50 mg/dL in women, or LDL-cholesterol surpassing 130 mg/dL. Hematological composite scores were computed as follows: neutrophil-to-lymphocyte ratio (NLR) = neutrophils/lymphocytes; monocyte-to-lymphocyte ratio (MLR) = monocytes/lymphocytes; platelet-to-lymphocyte ratio (PLR) = platelets/lymphocytes; systemic inflammation response index (SIRI) = neutrophils multiplied by monocytes, divided by lymphocytes. Neutrophils, monocytes, lymphocytes, and platelets were measured per 1000 cells/μL, except for platelets, which were measured per 100,000 cells/μL. Information regarding the therapies used in the disease was collected including the use of mesalazine, prednisone (as binary or mg/day), azathioprine and methotrexate, and biological therapies.

### 2.3. Statistical Analysis

In a previous work by our group, 430 patients with rheumatoid arthritis had a SIRI value of 1.22 ± 0.81 [8]. We have estimated that we would expect to find a difference in patients with IBD of 0.1. Thus, assuming an alpha error of 0.05 for a power of 80% and for an allocation between groups of 1:1, 194 subjects per group would have to be recruited. Demographic and clinical characteristics were presented as frequencies for binary variables. Continuous variable data were expressed as either mean ± standard deviation (SD) or as a median and interquartile range (IQR) for variables that did not follow a normal distribution. To assess univariate differences between patients and the control group, various statistical tests were employed, including Student’s *t*-test, the Mann–Whitney U-test, the chi-squared test, or Fisher’s exact test, depending on factors like distribution normality or sample size. Differences between IBD patients and controls in terms of hematological scores were examined using multivariable linear regression analysis, with the control group as the reference category. Confounding variables were selected from demographic factors and traditional cardiovascular risk factors if their p-values were less than 0.20 in the univariate analysis comparing patients and controls. All statistical analyses were carried out utilizing Stata software, version 17/SE (StataCorp, College Station, TX, USA), and a significance level of 5% was adopted for two-sided tests. A *p* value of less than 0.05 was considered indicative of statistical significance.

## 3. Results

### 3.1. Demographics and Disease-Related Data

A total of 197 IBD patients and 208 sex-matched controls with a mean ± SD age of 49 ± 10 and 50 ± 15 years, respectively, were included in this study. Demographic and disease-related characteristics of the participants are detailed in Table 1. The body mass index was higher in controls than in IBD patients (27 ± 5 vs. 29 ± 4 kg/m^2^, *p* ≤ 0.001). No significant differences were observed in smoking prevalence or dyslipidemia, but a higher proportion of controls had diabetes and hypertension. Among the patients, 66% had CD, and 32% had UC. The median disease duration for IBD was 12 years (IQR 8–19). In patients with CD, the predominant phenotypes were ileal and non-stricturing, non-penetrating. The median CDAI score was 39 (IQR 7–80), and 89% of the patients were classified as being in asymptomatic remission. Similarly, the Harvey–Bradshaw Index had a median score of 2 (IQR 0–4), with 82% of patients in the remission category based on this index. For UC, 52% had experienced pancolitis, and 78% had a partial Mayo score of less than 2 points. Further details concerning disease-related data can be found in Table 1.

### 3.2. Differences between Patients and Controls in Hematological Count Cells and Scores

Red cell, leucocyte and platelet count differences between patients with IBD and controls are shown in Table 2. Regarding red blood cells, although hemoglobin and hematocrit values did not differ between both groups, the mean corpuscular volume and the mean corpuscular hemoglobin were higher, and the mean corpuscular hemoglobin concentration was lower in IBD patients compared to controls after multivariable analysis. Furthermore, lymphocytes, eosinophils, and basophils were significantly lower in IBD patients compared to healthy controls after adjustment for covariates. However, platelets and the mean platelet volume did not show differences between patients and controls (Table 2). Regarding composite hematological scores, after multivariable analysis, NLR and PLR were higher in patients with IBD than in controls. This difference was not observed for SIRI and MLR. Similar results were found when this analysis was performed separately in patients with CD and UC (Supplementary Table S1). In this regard, NLR and PLR, but not SIRI or MLR, differed between patients with CD and healthy controls. Additionally, only PLR, but not SIRI, NLR or MLR, disclosed significant differences between patients with UC and healthy subjects (Supplementary Table S1).

### 3.3. Relationship of C-Reactive Protein, Fecal Calprotectin and Disease Activity Scores to Composite Hematological Scores

The relationship between CRP, fecal calprotectin and disease activity scores with composite blood-based scores is shown in Table 3. While CRP and SIRI and NLR were significantly and positively correlated, no relationship was found between CRP and MLR and PLR. Remarkably, fecal calprotectin did not disclose association with any of the hematological scores. In addition, concerning disease activity scores, CDAI score and Harvey-Bradshaw index, that correspond to CD, were not related to any composite blood scores. Similarly, partial Mayo score, that represents UC disease activity, was not significantly associated with the hematological scores (Table 3).

## 4. Discussion

In the present study we have analyzed four hematological scores, which have shown a relationship with certain outcomes in cancer, autoimmune, cardiovascular and inflammatory diseases, in a large series of patients with IBD. Based on our findings, NLR and PLR were significantly higher in IBD patients but this was not the case for SIRI and MLR. However, none of them showed a relationship with markers of systemic inflammation, fecal calprotectin or activity scores of both UC and CD.

In a previous work that evaluated SIRI in 87 patients with UC, patients were divided into active and non-active disease groups based on the Mayo score. In that study, SIRI was discovered to be superior in patients with active disease compared with UC in remission, and correlated with CRP [22]. Similarly, in a report of 187 patients with UC and 185 age- and sex-matched controls, higher SIRI levels were observed in moderate and severe UC subgroups compared to mild or remission subgroups [23]. Moreover, correlation analysis displayed that the SIRI levels were positively related with the Mayo score. This correlation maintained its significance after multivariable analysis. Similar findings were found in a work in 167 patients with UC and 106 controls [24]. SIRI significantly augmented in patients with UC and was closely correlated with the Mayo clinical score, Mayo endoscopic score, and Nancy histological index.

Regarding other blood-based scores, NLR and PLR have been found to be significantly elevated in UC subjects compared to controls in a report of 187 consecutive patients with UC and 185 age- and sex-matched healthy controls [23]. Similarly, in a work of UC patients in which 151 were active, and 36 in remission, NLR and PLR were significantly higher in the active group [25].

Few studies have assessed composite hematological scores in CD. In this regard, in a report of 44 patients with active CD, 66 patients with inactive CD, and 55 healthy blood donors, NLR values were found to be elevated in active CD compared to inactive CD patients plus controls, but no statistical differences were found between the active and inactive CD groups [26]. In contrast, in a report of 24 active and 25 inactive CD patients, the NLR was found to be higher in the active group [27].

Regarding MLR, a recent systematic review and meta-analysis that included nine studies found that MLR values were significantly higher in active IBD patients as compared to those under remission being these results consistent in both UC and CD patients with active disease [28]. However, our study does not support these previous findings. It should be noted that our sample size allowed us to perform multivariable analysis. Furthermore, the characterization of our series of patients has been broader than in previous works. On the other hand, in the studies discussed above, disease activity was generally dichotomized. In this regard, in our work we evaluated the scores in a continuous and binary manner. Moreover, it is important to take into account that the majority of patients from our series, both in UC and CD, had low disease activity. This would support the fact that these scores are not valid to measure disease activity in patients when the disease is under control.

In our study we found that patients with IBD had a BMI in the overweight range but significantly lower than controls. This is in line with previous studies in which it has been described that patients with IBD have lower BMI values compared to controls [29]. This is believed to be due to the inflammatory activity of the disease. However, we believe this fact may have not affected our results since the differences in hematological scores between patients and controls were adjusted for this BMI.

We acknowledge the limitation that for UC subjects, partial Mayo score and not complete Mayo score was available for these patients. Furthermore, the cross-sectional design of our work prevents concluding causality. Additionally, patients with IBD may present hematological disruptions caused by certain therapies used in the disease such as methotrexate or azathioprine.

## 5. Conclusions

In conclusion, the SIRI, NLR, MLR and PLR hematological-based scores are not appropriate for the monitoring of disease activity in patients with IBD that are in the range of low or moderate activity.

## Figures and Tables

**Table 1:** Characteristics of patients with inflammatory bowel disease and controls.

	Controls	IBD Patients	
	(n = 208)	(n = 197)	*p*
Age, years	50 ± 15	49 ± 10	0.25
Female, n (%)	124 (59)	107 (54)	0.28
Body mass index, kg/m^2^	**29 ± 4**	**27 ± 5**	**<0.001**
Abdominal circumference, cm	93 ± 8	94 ± 12	0.49
Cardiovascular co-morbidity			
Smoking, n (%)	45 (22)	39 (20)	0.65
Diabetes, n (%)	**29 (14)**	**11 (6)**	**0.004**
Hypertension, n (%)	**63 (30)**	**35 (18)**	**0.003**
Dyslipidemia, n (%)	190 (77)	157 (80)	0.53
Obesity, n (%)	57 (27)	55 (28)	0.91
Statins, n (%)	**47 (23)**	**21 (11)**	**0.001**
IBD related data			
Ulcerative colitis, n (%)		67 (34)	
Crohn’s disease, n (%)		130 (66)	
Disease duration since diagnosis, years		12 (8-19)	
CRP, mg/L	2.0 (1.0–4.8)	1.8 (0.9–3.8)	0.30
Ulcerative Colitis related data, n (%)			
Partial Mayo score		1 (0-1)	
<2		52 (78)	
≥2		15 (21)	
Pancolitis		34 (52)	
Left-sided colitis		23 (35)	
Proctosigmoiditis		7 (10)	
Crohn’s Disease related data, n (%)			
A1 below 16 years		19 (15)	
A2 between 17 and 40 years		81 (62)	
A3 above 40 years		27 (21)	
B1 non-stricturing, non-penetrating		73 (56)	
B2 stricturing		46 (35)	
B3 penetrating		14 (11)	
L1 ileal		56 (43)	
L2 colonic		23 (18)	
L3 ileocolonic		51 (39)	
L4 isolated upper disease		11 (8)	
Harvey-Bradshaw Index		2 (0–4)	
Clinical remission		106 (82)	
Mildly active disease		14 (11)	
Moderately active disease		8 (6)	
Severely active disease		1 (1)	
CDAI score		39 (7–80)	
Asymptomatic remission		116 (89)	
Mildly to moderately active Crohn disease		10 (8)	
Moderately to severely active Crohn disease		3 (2)	
Severely active to fulminant disease		0 (0)	
Fecal calprotectin, mcg/g		113 (30–251)	
>150		96 (49)	
≥150		71 (36)	
Perianal disease, n (%)		23 (12)	
Previous surgery, n (%)		55 (28)	
Oral mesalazine, n (%)		175 (89)	
Prednisone, mg/day		8 (5–20)	
Current prednisone, n (%)		6 (2)	
Methotrexate, n (%)		22 (11)	
Azathioprine, n (%)		61 (31)	
Anti-TNF therapy, n (%)		58 (29)	
Ustekinumab, n (%)		8 (4)	
Vedolizumab, n (%)		5 (3)	
Tofacitinib, n (%)		4 (2)	

Data represent mean ± SD or median (interquartile range) when data were not normally distributed. BMI: body mass index; CRP: C reactive protein; TNF: tumor necrosis factor. CDAI was categorized as 0 to 149: asymptomatic remission; 150 to 220 points: mildly to moderately active; 221 to 450 points: moderately to severely active; 451 to 1100 points: severely active to fulminant disease. The Harvey-Bradshaw Index was categorized as 0 to 4 points: clinical remission; 5 to 7 points: mildly active disease; 8 to 16 points: moderately active disease; 17 to 100 points: severely active disease. Dyslipidemia was characterized by meeting any of the following criteria: total cholesterol exceeding 200 mg/dL, triglyceride levels surpassing 150 mg/dL, HDL-cholesterol below 40 mg/dL in men or under 50 mg/dL in women, or LDL-cholesterol exceeding 130 mg/dL. Significant *p* values are depicted in bold.

**Table 2:** Multivariable analysis of the differences between patients and controls in hematological count cells and scores.

	Controls	IBD Patients			
	(n = 208)	(n = 197)	*p*	Beta Coef. (95%CI)	*p*
	Univariable			Multivariable	
Red blood cells, ×10^6^/mm^3^	4.76 ± 0.49	4.67 ± 0.47	0.056	−0.09 (−0.19–0.01)	0.076
Hemoglobin, g/dL	14.0 ± 1.5	14.0 ± 1.5	0.77		
Hematocrit, %	42.9 ± 4.1	42.7 ± 3.9	0.63		
Mean corpuscular volume, fL	**90.3 ± 5.8**	**91.7 ± 5.6**	**0.011**	**1.6 (0.5–2.8)**	**0.006**
Mean corpuscular hemoglobin, pg	**29.6 ± 2.5**	**30.3 ± 2.6**	**0.003**	**0.8 (0.3–1.3)**	**0.003**
Mean corpuscular hemoglobin concentration, g/dL	**32.7 ± 1.2**	**30.9 ± 5.8**	**<0.001**	**−1.6 (−2.5–(−0.8))**	**<0.001**
Leucocytes/mm^3^	**7480 ± 1941**	**7003 ± 2079**	**0.019**	−292 (−701–118)	0.16
Neutrophils/mm^3^	4154 ± 1504	4139 ± 1615	0.92		
Lymphocytes/mm^3^	**2427 ± 827**	**2037 ± 835**	**<0.001**	**−339 (−509–(−169))**	**<0.001**
Monocytes/mm^3^	600 ± 171	584 ± 249	0.48		
Eosinophils/mm^3^	**245 ± 176**	**196 ± 169**	**0.005**	**−44 (−80–(−8))**	**0.016**
Basophils/mm^3^	**50 ± 26**	**43 ± 25**	**0.006**	**−6 (−11–(−1))**	**0.027**
Platelets ×10^3^/mm^3^	263 ± 59	270 ± 69	0.27		
Mean platelet volume, fL	10.2 ± 0.9	10.2 ± 1.0	0.69		
Composite hematological scores					
Systemic inflammation response index (SIRI) ×10^−3^	1. 23 ± 1.20	1.36 ± 0.94	0.26		
Neutrophil-to-lymphocyte ratio	**1.99 ± 1.57**	**2.32 ± 1.24**	**0.022**	**0.3 (0.03–0.6)**	**0.033**
Monocyte-to-lymphocyte ratio	0.29 ± 0.23	0.34 ± 0.38	0.085	0.05 (−0.01–0.1)	0.13
Platelet-to-lymphocyte ratio	**125 ± 79**	**156 ± 80**	**<0.001**	**27 (11–44)**	**0.001**

In the multivariable analysis controls is considered the reference category. IBD: Inflammatory bowel disease. Multivariable analysis is adjusted for body mass index, diabetes, hypertension, and statins intake. Significant *p* values are depicted in bold.

**Table 3:** Relationship of C-reactive protein, fecal calprotectin and disease activity scores to composite hematological scores.

	Beta Coef. (95%CI), *p*							
	SIRI		NLR		MLR		PLR	
CRP, mg/L	**55 (25–84)**	**<0.001**	**0.06 (0.02–0.1)**	**0.003**	0.007 (−0.01–0.01)	0.91	1 (−1–4)	0.28
Fecal calprotectin, mcg/g	0.1 (−0.2–0.4)	0.44	0.00005 (−0.0004–0.0005)	0.82	3 × 10^−6^ (−0.0001–0.0001)	0.97	0.009 (−0.02–0.03)	0.50
Crohn’s disease								
CDAI score	0.6 (−2–3)	0.65	0.001 (−0.002–0.004)	0.50	−0.0004 (−0.001–0.0008)	0.52	0.06 (−0.1–0.3)	0.54
Asymptomatic remission	ref.		ref.		ref.		ref.	
Mildly to moderately active	309 (−372–991)	0.37	0.4 (−0.5–1.3)	0.35	0.02 (−0.3–0.3)	0.89	17 (−40–73)	0.56
Moderately to severely active	−97 (−1246–1054)	0.87	−0.6 (−2.1–0.9)	0.40	−0.1 (−0.6–0.4)	0.66	−23 (−118–71)	0.63
Harvey-Bradshaw Index	6 (−53–65)	0.84	0.009 (−0.07–0.08)	0.83	−0.02 (−0.04–0.01)	0.21	−3 (−8–1)	0.16
Clinical remission	ref.		ref.		ref.		ref.	
Mildly active disease	−32 (−595–531)	0.91	−0.3 (−1.1–0.4)	0.38	−0.08 (−0.3–0.2)	0.52	−44 (−89–1)	0.056
Moderately to severity active	−72 (−797–652)	0.84	−0.1 (−1.0–0.9)	0.90	−0.1 (−0.5–0.2)	0.44	−46 (−104–12)	0.12
Ulcerative Colitis related data								
Partial Mayo score	−3 (−138–133)	0.97	0.03 (−0.2–0.2)	0.73	−0.008 (−0.3–0.01)	0.50	−2 (−15–11)	0.79
<2	ref.		ref.		ref.		ref.	
≥2	−48 (−535–438)	0.84	0.2 (−0.4–0.9)	0.52	−0.01 (−0.09–0.07)	0.73	3 (−43–49)	0.91

Hematological scores are the dependent variables in this analysis. CDAI was categorized as 0 to 149: asymptomatic remission; 150 to 220 points: mildly to moderately active; 221 to 450 points: moderately to severely active; 451 to 1100 points: severely active to fulminant disease. The Harvey-Bradshaw Index was categorized as 0 to 4 points: clinical remission; 5 to 7 points: mildly active disease; 8 to 16 points: moderately active disease; 17 to 100 points: severely active disease. Significant *p* values are depicted in bold.

## Data Availability

The data sets used and/or analyzed in the present study are available from the corresponding author upon request.

## Supplementary Materials

The following supporting information can be downloaded at: https://www.mdpi.com/article/10.3390/jcm12237248/s1, Table S1: Univariable analysis of the differences between patients with Crohn’s disease and ulcerative colitis and controls in hematological count cells and scores.
