# Supplementary material for: Hematological Composite Scores in Patients with Inflammatory Bowel Disease
Source: J Clin Med. 2023 Nov 23;12(23):7248. doi: 10.3390/jcm12237248 (PMC10706900; doi:10.3390/jcm12237248)
Supplement: Supplementary file 1 [file jcm-12-07248-s001.zip › jcm-2716580-supplementary.pdf]

Supplementary Table S1. Univariable analysis of the differences between patients with Crohn's disease and ulcerative colitis and controls in hematological count cells and scores.

	Controls (n=208)	Chron's disease (n=130)	p*	Ulcerative colitis (n=67)	p**
Red blood cells, x10e6/mm3	4.76 ± 0.49	4.66 ± 0.46	0.057	4.69 ± 0.50	0.30
Hemoglobin, g/dL	14.0 ± 1.5	14.0 ± 1.5	0.81	14.0 ± 1.41	0.81
Hematocrit, %	42.9 ± 4.1	42.6 ± 3.7	0.59	42.8 ± 4.1	0.87
Mean corpuscular volume, fL	**90.3 ± 5.8**	**91.2 ± 6.1**	**0.017**	**91.5 ± 4.5**	**0.13**
Mean corpuscular hemoglobin, pg	**29.6 ± 2.5**	**30.5 ± 2.8**	**0.002**	**30.0 ± 2.0**	**0.17**
Mean corpuscular hemoglobin concentration, g/dl	**32.7 ± 1.2**	**30.6 ± 6.4**	**<0.001**	**31.5 ± 4.7**	**0.001**
Leucocytes / mm3	7480 ± 1941	7142 ± 2157	0.14	**6748 ± 1920**	**0.008**
Neutrophils / mm3	4154 ± 1504	4301 ± 1722	0.42	3840 ± 1358	0.13
Lymphocytes / mm3	**2427 ± 827**	**2010 ± 816**	**<0.001**	**2089 ± 871**	**0.004**
Monocytes / mm3	600 ± 171	592 ± 273	0.74	571 ± 197	0.26
Eosinophils / mm3	245 ± 176	189 ± 161	0.004	209 ± 185	0.15
Basophils / mm3	50 ± 26	41 ± 23	0.002	47 ± 29	0.38
Platelets x10e3 / mm3	263 ± 59	268 ± 67	0.24	269 ± 73	0.38
Mean platelet volume, fL	10.2 ± 0.9	10.3 ± 1.0	0.28	10.1 ± 1.1	0.42
Composite hematological scores					
SIRI x 10e-3	1. 23 ± 1.20	1.44 ± 0.81	0.12	11.21 ± 8.25	0.88
NLR	**1.99 ± 1.57**	**2.44 ± 1.28**	**0.008**	2.10 ± 1.13	0.61
MLR	0.29 ± 0.23	0.36 ± 0.46	0.055	0.31 ± 0.14	0.55
PLR	**125 ± 79**	**160 ± 81**	**<0.001**	**150 ± 78**	**0.025**

In this analysis controls is considered the reference category. Significant p values are depicted in bold.
p* refers to the comparison between Chron's disease and controls, and p** to the comparison between ulcerative colitis and controls.
SIRI: systemic inflammation response index; NLR: neutrophil-to-lymphocyte ratio; PLR: platelet-to-lymphocyte ratio; MLR: monocyte-to-lymphocyte ratio.
